# Supplementary material for: A high-resolution mRNA expression time course of embryonic development in zebrafish
Source: eLife. 2017 Nov 16;6:e30860. doi: 10.7554/eLife.30860 (PMC5690287; doi:10.7554/eLife.30860)
Supplement: Supplementary file 6. [file elife-30860-supp6.zip › biolayout-clusters-files/Cluster215-genes.html]

Cluster215


# Cluster215: Genes

| Ensembl ID | Gene Name | Chr | Start | End | Biotype |
| --- | --- | --- | --- | --- | --- |
| ENSDARG00000079363 | LRIG3 | 15 | 2978599 | 3011650 | protein\_coding |
| ENSDARG00000069185 | celsr1a | 4 | 28202325 | 28346056 | protein\_coding |
| ENSDARG00000091481 | foxc1a | 2 | 814507 | 816681 | protein\_coding |
| ENSDARG00000079248 | mn1b | 5 | 65997251 | 66023374 | protein\_coding |
| ENSDARG00000103129 | nid1b | 11 | 44286305 | 44341597 | protein\_coding |
| ENSDARG00000012574 | slkb | 1 | 48962098 | 49004702 | protein\_coding |
| ENSDARG00000035563 | znf703 | 5 | 23826183 | 23829884 | protein\_coding |
